# Transactions of the Medical Society of the State of New York, Vol. VII, Part II

**Published:** 1848-06

**Authors:** 


*Transactions of the Medical Society of the State of New York. Vol. VII, Part II.*

The Transactions of the State Society for the present year embrace an address before the society, by Dr. Thomas W. Blatchford, M. D., on the temperaments; *History of the diseases of Saratoga county,* by Dr. Oliver Brisban; address delivered before the Medical Society of the County of Columbia, by Joseph Bates, M. D., on *the influence of the mind on disease; observations on agriculture in its bearings on medicine,* by Alexander Thomson M, D.; *on the influence of dress in the production of disease iu Females*, read before the Chenango county Medical Society, by Dr. Wm. D. Purple. They are all interesting and creditable productions.

In the appendix, containing an abstract of the proceedings, we notice the following items:—

"By invitation, Dr. Clarke, of New York, addressed the Society on the subject of fevers, advocating the doctrine of the contagiousness of typhus and ship fevers, and the contagiousness of bilious remittent fevers."

Among some resolutions accepted and referred to a committee, is the following: — "In the opinion of this society, every Physician who would discharge with fidelity the duties of his high vocation, must neccessarily avail himself of some of the principal periodicals, as well as the privilege of frequent association with his brethren of the profession."

The committees on the subjects of hygiene, Lunatic Assylum, &c., did not report, and were continued.

The code of ethics adopted by the National Convention was adopted as the code of the Society.

The report of the National Medical Convention on the subject of preliminary education was approved, and it was reccommended that County Societies act accordingly, ( action has already been taken on this subject by the Medical Association of this city.)

The almost unanimous opinion of the members present was, that fevers do not now require or bear bleeding, as in former years.

Dr. Van Buren related a case of croup in which a sponge saturated with sol. nitras agenti was applied " thoroughly to the upper part of the trachea, and adjacent parts!! "

It was resolved that "the identity or non-identity of typhus and typhoid fever, be referred for discussion to the Society at its meeting in 1849.— Also, for discussion at the same time, croup and its kindred affections."

The following gentlemen were elected honorary members—Drs. Woodbridge Strong, of Boston, Frederick May, Washington city. Dr. Strong is the author of the article on the treatment of typhus fever, of which we gave some account in a recent number of this journal. We trust the honor now conferred upon him may lead him to think more favorably of his professional brethren. Our reader may recollect that in the article referred to he characterised them as *second-rate nurses.*

